# Physical activity and sedentary behavior during pregnancy and postpartum, measured using hip and wrist-worn accelerometers

**DOI:** 10.1016/j.pmedr.2018.04.012

**Published:** 2018-04-19

**Authors:** Kathryn R. Hesketh, Kelly R. Evenson, Marissa Stroo, Shayna M. Clancy, Truls Østbye, Sara E. Benjamin-Neelon

**Affiliations:** a Department of Health, Behavior and Society, Johns Hopkins Bloomberg School of Public Health, Baltimore, MD, USA; b CEDAR and MRC Epidemiology Unit, University of Cambridge, Cambridge, UK; c Department of Epidemiology, Gillings School of Global Public Health, University of North Carolina, Chapel Hill, NC, USA; d Department of Community and Family Medicine, Duke University Medical Center, Durham, NC, USA

**Keywords:** US, United States, SED, Sedentary time, LPA, Light physical activity, MPA, Moderate physical activity, VPA, Vigorous physical activity, MVPA, Moderate-to-vigorous physical activity, VM, Vector magnitude, T2, Trimester 2, T3, Trimester 3, PP3, 3 months postpartum, PP6, 6 months postpartum, PP9, 9 months postpartum, PP12, 12 months postpartum, Physical activity, Pregnancy, Postpartum, Sedentary behavior, Measurement

## Abstract

**Background:**

Physical activity in pregnancy and postpartum is beneficial to mothers and infants. To advance knowledge of objective physical activity measurement during these periods, this study compares hip to wrist accelerometer compliance; assesses convergent validity (correlation) between hip- and wrist-worn accelerometry; and assesses change in physical activity from pregnancy to postpartum.

**Methods:**

We recruited women during pregnancy (*n* = 100; 2014–2015), asking them to wear hip and wrist accelerometers for 7 days during Trimester 2 (T2), Trimester 3 (T3), and 3-, 6-, 9- and 12-months postpartum. We assessed average wear-time and correlations (axis-specific counts/minute, vector magnitude counts/day and step counts/day) at T2, T3, and postpartum.

**Results:**

Compliance was higher for wrist-worn accelerometers. Hip and wrist accelerometers showed moderate to high correlations (Pearson's r 0.59 to 0.84). Hip-measured sedentary and active time differed little between T2 and T3. Moderate-to-vigorous physical activity decreased at T3 and remained low postpartum. Light physical activity increased and sedentary time decreased throughout the postpartum period.

**Conclusions:**

Wrist accelerometers may be preferable during pregnancy and appear comparable to hip accelerometers. As physical activity declines during later pregnancy and may not rebound post birth, support for re-engaging in physical activity earlier in the postpartum period may benefit women.

## Highlights

• Wrist-worn accelerometers are suitable for physical activity measurement in pregnancy.
• Wrist-worn (vs. hip-worn) accelerometers provide comparable physical activity estimates.
• Total/moderate-vigorous physical activity (MVPA) declines in later pregnancy.
• Light physical activity appears to increase and MVPA decrease after birth.
• Support may help women re-engage in M/VPA earlier in postpartum.

## 1 Introduction

Physical activity confers benefits to physical and mental health (Department of Health, 2011; Department of Health and Human Services, 2008), including during pregnancy and the postpartum period (American College of Obstetricians and Gynecologists, 2015). In women with uncomplicated pregnancies, regular physical activity is known to facilitate weight management and physical fitness, reduce the risk of gestational diabetes, and improve mental wellbeing (American College of Obstetricians and Gynecologists, 2015). The American College of Obstetrics and Gynecology (American College of Obstetricians and Gynecologists, 2015) therefore advocates that women engage in at least 20–30 min of exercise on most or all days of the week, with activity ideally spread throughout the week. Though women should be aware of medical contraindications (American College of Obstetricians and Gynecologists, 2015), those who were physically active prior to pregnancy can continue to be so, and those who lead more sedentary lives also benefit from gradual increases in physical activity (American College of Obstetricians and Gynecologists, 2015; U.S. Department of Health and Human Services, 2008).

To date, much of the research assessing women's physical activity during pregnancy and postpartum, including derivation of the guidelines, has relied on self-report measures (da Silva et al., 2016). Studies conducted in nationally representative samples of women in the United States (US) indicate that many women self-report engaging in low levels of physical activity during pregnancy and do not meet physical activity guidelines (Evenson and Wen, 2010; Hesketh and Evenson, 2016). Further, a review of studies in pregnant women showed that the agreement between questionnaire derived physical activity levels and objective measures (i.e. pedometers and accelerometers) was only slight to fair (Evenson et al., 2012a). A limited number of epidemiological studies that have used objective measures (e.g., accelerometer) to assess physical activity during pregnancy, suggest that physical activity is indeed even lower compared to that self-reported by women (da Silva et al., 2016).

Objective measures of physical activity provide insight in addition to self-report measures as they minimize responder and recall bias, providing a more tangible estimate of frequency, duration, and intensity of women's physical activity. Objective accelerometer placement has, to date, tended to be at the hip, with most epidemiological studies to date using hip-worn accelerometers to measure physical activity (da Silva et al., 2016). However, there has been a recent move toward using wrist-worn activity accelerometers in larger epidemiological studies (Mannini et al., 2013). Wrist-worn accelerometers may be more convenient to wear and encourage greater compliance with wear protocols (Mannini et al., 2013). They may also be particularly well-suited to measuring physical activity during pregnancy given the potential practical difficulties of wearing a hip-worn accelerometer. Comparison of the two placement locations, using the same monitor type, has been conducted, for example in younger (Tudor-Locke et al., 2015) and older adults (Kamada et al., 2016), but, to our knowledge, not in pregnant women.

A recent study evaluated validity and reliability of accelerometry in pregnancy and postpartum with accelerometers placed on the hip, ankle, and triceps, but this was a lab-based study (Conway et al., 2018). Indeed, assessing the correlation between locations in free-living situations is scarce, and a large proportion of evidence about physical activity during pregnancy and postpartum therefore comes from subjective self-report measures. Moreover, with the known benefits of being active for both mother and child, it is important to establish valid and realistic high-compliance protocols for assessing physical activity and sedentary behavior using objective measures during this important period.

Although relatively few studies have assessed how physical activity and sedentary behavior changes from pregnancy to postpartum, several cohort studies have used self-report measures to suggest that there is an overall decrease in physical activity during pregnancy (Borodulin et al., 2008; McParlin et al., 2010; Rousham et al., 2006) and subsequent rebound and maintenance postpartum (Borodulin et al., 2008; Borodulin et al., 2009; Melzer et al., 2009; Pereira et al., 2007). This is borne out in several studies using objective measures: both a UK and Norwegian study suggest that women's moderate-to-vigorous physical activity (MVPA) decreases during pregnancy (Currie et al., 2015; Richardsen et al., 2016), but may then increase after delivery (Richardsen et al., 2016). In a sample of 80 women from North Carolina, at both 3 and 12 months postpartum women engaged in approximately 20 min/day of MVPA (Evenson et al., 2012b). Women's average counts per minute (cpm) did, however, increase from 3- to 12-months postpartum, indicating an increase in total physical activity. Decreases in sedentary time were also observed over the same period (9.3 h to 8.8 h per day) (Evenson et al., 2012b).

This study therefore sought to assess physical activity and sedentary behavior in a sample of low-resource women during pregnancy and postpartum, using wrist- and hip-worn accelerometers. Specifically, the aims of this paper were to: a) determine the relative wear-time and compliance with each accelerometer type, b) assess the convergent validity (or correlation) between hip and wrist accelerometers at several time points during pregnancy and postpartum; and c) using hip-worn accelerometers, determine how physical activity and sedentary behavior changes during pregnancy and postpartum. It was hypothesized that women would have greater compliance with wrist-worn accelerometers, and that physical activity would decrease and sedentary behavior would increase during pregnancy but rebound during the year following birth.

## 2 Methods

### 2.1 Study participants

We used data from Nurture, a US cohort study of low-income, predominately black mother-child pairs followed from pregnancy to 12 months postpartum. The study is described in detail elsewhere (Neelon et al., 2017); briefly, we enrolled 860 women during pregnancy, of whom 799 delivered a singleton live infant, and 666 were eligible to participate at 3 months postpartum. The purpose of Nurture was to determine the influence of multiple caregivers on infant anthropometric outcomes and health behaviors in the first year of life (Neelon et al., 2017). Women were eligible to participate in Nurture if they were 20–36 weeks pregnant; carrying a singleton with no known congenital abnormalities; were ≥18 years of age; could speak and read English; intended to keep the baby; and planned to stay within the area until at least 12 months postpartum (Neelon et al., 2017).

For these analyses, we used data from a convenience sample of women who consented to participate in the sub-study to assess how objectively-measured physical activity during pregnancy and postpartum was related to weight gain and subsequent weight retention. From September 2014, we asked all participants already enrolled in the Nurture study, who were still pregnant (between 20.0 and 35.0 weeks), if they wanted to participate in the sub-study during a prenatal visit. We also invited new participants in Nurture to participate in the sub-study after they had provided informed consent for the primary study. Study staff gave all women a full explanation of study procedures and showed them the accelerometers; we then obtained informed consent, separately from the Nurture study, if women wanted to participate. We ceased recruitment when 100 women had consented to participate. We collected data from September 2014 through April 2015. The Duke University Medical Center Institutional Review Board (Pro 00036242) provided ethical approval for the main Nurture study and physical activity sub-study.

### 2.2 Data collection

We approached 167 women to participate in the sub-study. At recruitment [during trimester 2 (T2) (*n* = 42) or 3 (T3) (*n* = 58)], we asked women to wear two ActiGraph GT3X+ accelerometers (Pensacola, Florida, USA; valid and reliable in adult women (McClain et al., 2007; Ozemek et al., 2014; Sasaki et al., 2011) and specifically, in pregnant and postpartum women (Conway et al., 2018)) for 7 days to measure free-living physical activity and sedentary behavior. Women wore one accelerometer on the wrist for 24 h/day and, concurrently, one at the hip during waking hours only (removing accelerometers during water-based activity or bathing). We asked those recruited in T2 to also wear the accelerometers again during T3 (*n* = 31). At baseline (either T2 or T3), we asked women to complete a questionnaire to gather socio-demographic and pre-pregnancy anthropometric data. We subsequently contacted women at 3-, 6,- 9-, and 12-months post birth (PP3, PP6, PP9 and PP12 respectively) and asked those who had participated in the sub-study to follow the same physical activity measurement protocol.

### 2.3 Physical activity data

For both accelerometer locations, we collected physical activity data in 60-second epochs. We downloaded and processed these data using ActiLife software (ActiGraph, Pensacola, FL, USA). We defined accelerometer non-wear time as an interval of ≥90 consecutive minutes of zero counts/min, allowing up to 2 min of nonzero counts if no counts were detected during the 30 min up- or downstream of that interval (Choi et al., 2011). Any nonzero counts (except the allowed short intervals) were considered wear time. As done previously in older women (Kamada et al., 2016), for both the hip and wrist accelerometers we extracted three types of data derived through ActiLife: three axis-specific counts/min (cpm), total number of steps/day, and vector magnitude counts/day. We removed data between 12:00 midnight and 6:00 am for both sets of accelerometers, as this was when women were indicated to usually be asleep based on nonwear of the hip accelerometer. Sensitivity analyses, using activity diaries completed by a number of women, indicated that our pre-defined non-wear period corresponded (on average, within one hour) to the period when women reported being asleep. We considered women with at least 3 days of measurement (≥10 h/day for the wrist (Troiano et al., 2008), ≥8 h/day for the hip (Evenson and Terry, 2009)) to have valid data for inclusion.

For hip accelerometers only, we analyzed cpm data to derive minutes of sedentary, light (LPA), moderate (MPA), vigorous (VPA), and MVPA women engaged in at each time point. We defined sedentary time as <100 cpm (Matthews et al., 2008). We applied two sets of cut points commonly applied to women of childbearing age to define physical activity [Troiano: LPA: 100–2019 cpm, MPA/MVPA: 2020–5998 cpm, VPA:>5999 cpm (Troiano et al., 2008); and Swartz: LPA: 100–573 cpm, MPA/MVPA: 574–4944 cpm, VPA:≥4945 cpm^30^].

### 2.4 Demographic variables

We derived a number of socio-demographic variables for descriptive purposes. Maternal age (in years), ethnicity and race, and pre-pregnancy weight and height were reported at baseline. We used weight and height to derive pre-pregnancy body mass index (BMI) (kg/m^2^) and categorize women as under (<18.5), normal (18.5–24.9), overweight (25.0–29.9), or obese (≥30.0) using World Health Organisation (2013) classifications. Women also reported their pre-pregnancy household income [less than or equal to $20,000; $20,001 to $40,000; more than $40,000]; highest level of educational attainment [high school/graduate; some college; advanced/college degree]; employment status [employed; unemployed (looking for work); unemployed (not looking for work)]; relationship status [married; not married, living with partner; never married; divorced]; number of children in the home (before the birth of the cohort child) [0; 1; 2/+]; and if they had ever smoked cigarettes or used tobacco [yes/no].

### 2.5 Statistical analysis

We carried out analyses using STATA/SE 14 (StataCorp, 2014). We included all women meeting the validity criteria (i.e., both valid hip and wrist data at least one time point during pregnancy, with at least 3 days of measurement (≥10 h/day for the wrist (Troiano et al., 2008)), ≥8 h/day for the hip (Evenson and Terry, 2009)). We calculated descriptive sample characteristics and average wear time for hip- and wrist-worn accelerometers to compare compliance between the two wear locations; a significance level of 0.05 was set a priori.

As data were aggregated at the hourly level, we matched hip and wrist data by hour, day of measurement, and participant identification number; therefore, we included only valid wear-time when both accelerometers were being worn in the correlation analyses. At each time point for each woman, we derived three measures for total daily averages for hip and wrist: counts; VM counts; and step counts. We conducted Pearson's test for correlation to assess the convergent validity between each of the three types of data during T2, T3 and postpartum (aggregating measures for PP3-PP12 to ensure sufficient numbers). We also grouped participants into quartiles (ensuring equal numbers in each of the four groups) based on the total VM counts/day and steps/day for hip and wrist accelerometers. We calculated overall percentage agreement as the proportion of participants who were in the same quartile for outcomes measured at both wear locations, evaluated using a weighted Kappa statistic. Following Landis and Koch (1977) a kappa <0.40 indicates fair agreement, 0.41 and 0.60 moderate, 0.61–0.80 substantial and values >0.81 almost perfect agreement.

To assess how physical activity and sedentary behavior changed over time, we used hip-worn accelerometer data only to explore two-level random intercept regression models (i.e., how total minutes of physical activity at each intensity/outcome differed across the measurement period; daily minutes spent in sedentary, in LPA, and in MVPA, and VM and step counts). Hierarchical models allowed for variation in outcome between days (level 1) and variation between women (level 2), with correlations between observations accounted for by allowing the intercept to vary randomly between women (i.e., level 2). Time was entered into the model as a main effect, with T3 as the baseline. Models were also run with T2 and PP3 as the baseline time point. All models were adjusted for daily wear-time. Women who provided at least 3 valid days of hip-worn physical activity data at one time point during pregnancy and one time point postpartum were included in analyses. We also explored how using two differing cut points to derive women's daily minutes of physical activity (Swartz et al., 2000; Troiano et al., 2008) influenced our findings.

We conducted sensitivity analyses adjusting models for descriptive variables (maternal age (in years), ethnicity, pre-pregnancy BMI, household income, educational attainment, employment status, relationship status, number of children at home, ever smoked tobacco). This changed regression coefficients very little and did not alter the significance of associations. We therefore present the minimally adjusted models (wear-time only) as extensive adjustment may constitute over adjustment here.

## 3 Results

Women recruited into the sub-study were broadly representative of the full Nurture cohort, being predominately black, with low educational attainment, and two-thirds had an annual household income of ≤$20,000 (Table 1) (Neelon et al., 2017). One hundred women provided valid written consent, of whom 87 provided any data during pregnancy and 55 subsequently provided physical activity data postpartum. There were no significant demographic differences (for characteristics listed in Table 1) between women who only provided consent (*n* = 13), provided physical activity data during pregnancy (*n* = 87) or provided physical activity data postpartum (*n* = 55).

**Table 1:** Characteristics of women consented into and participating in the nurture sub-study.

	Consented (*n* = 100)		Pregnancy^a^ (*n* = 87)		Postpartum^a^ (*n* = 55)	
	*n*	%	*n*	%	*n*	%
Maternal ethnicity: Hispanic/Latina	8	8	6	7	2	4
Maternal race						
Black/African American	76	76	68	77	42	77
White	16	16	14	16	9	16
Other^b^/unknown	8	8	6	6	4	6
Age at recruitment^c^						
≤20 years	11	11	5	6	3	6
21–30 years	67	67	61	74	36	71
31–40 years	22	22	17	20	12	24
Maternal education (highest completed)						
High school/graduate	47	48	44	51	27	50
Some college	44	44	36	41	22	41
Advanced/degree	8	8	7	8	5	9
Maternal employment status						
Employed	51	51	45	52	28	50
Unemployed (seeking work)	14	14	10	11	9	16
Unemployed (not seeking work)	35	35	33	38	18	33
Household income						
Less than or equal to $20,000	65	66	56	65	34	64
$20,001 to $40,000	21	21	19	22	12	23
More than $40,000	12	12	11	13	7	13
Relationship status						
Married	19	19	17	20	14	25
Not married, living with partner	37	38	33	38	19	35
Never married	34	35	30	35	18	33
Divorced	8	8	6	7	4	7
Children in the home						
0	28	29	25	29	19	35
1	36	37	31	35	15	28
2/+	34	35	31	35	20	37
Pre-pregnancy BMI						
Underweight	9	9	6	7	2	4
Normal weight	31	31	29	33	14	26
Overweight	22	22	22	25	17	31
Obese	37	37	30	34	21	39
Ever smoked tobacco						
Yes	48	49	42	49	26	48
No	50	51	44	51	28	52

a Number of women who were consented and then provided physical activity data during pregnancy (T2 and/or T3), and subsequently postpartum (1 or more at 3-, 6-, 9- or 12-months).

b Includes 5 Native American and 3 other/unknown race.

c Age range 18–40 years.

### 3.1 Accelerometer compliance

Women wore hip accelerometers for a mean 12.6 (SD: 2.2) hours across 6.5 (1.7) days in T2 and 12.3 (2.1) hours across 6.2 (1.8) days in T3. In the postpartum period, women wore hip accelerometers for a mean 13.1 (1.8) hours across 5.7 (1.7) days. Women provided greater amounts of adherent data from wrist-worn accelerometers, which remained consistent across pregnancy and postpartum [T2: mean 16.3 (2.0) hours across 7.6 (1.3) days; T3: 16.2 (1.9) hours across 7.4 (1.6) days; Postpartum 16.2 (1.9) hours across 7.4 (1.5) days].

### 3.2 Correlation and agreement between hip- and wrist-worn accelerometers

**Fig. 1:**
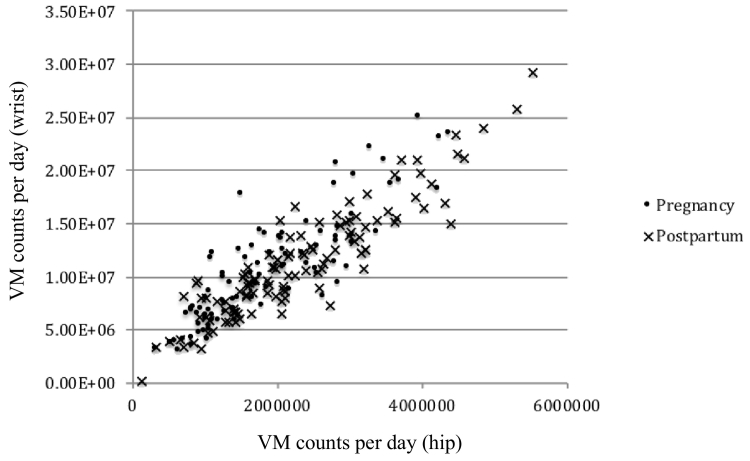
Correlation between hip- and wrist-worn vector magnitude (VM) counts/day during pregnancy and postpartum in the Nurture study (North Carolina, 2014–2015, *n* = 43).

**Fig. 2:**
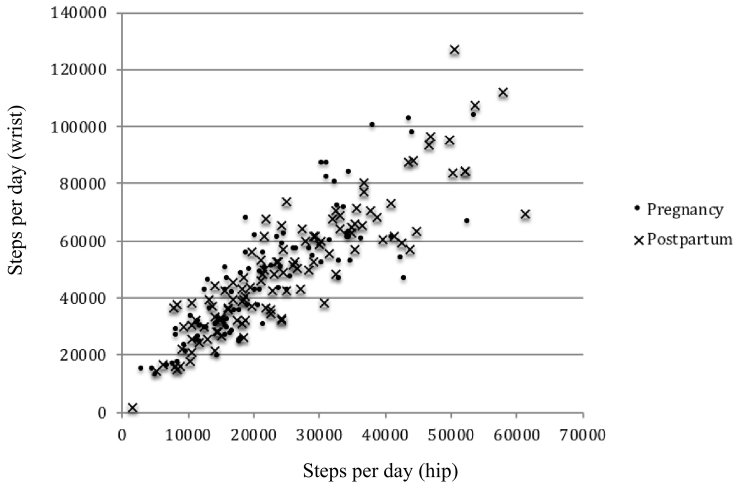
Correlation between hip- and wrist-worn steps/day during pregnancy and postpartum in the Nurture study (North Carolina, 2014–2015, *n* = 43).

Correlations between hip and wrist-worn accelerometers in T2, T3, and postpartum are shown in Table 2, Fig. 1 and Fig. 2. Axis-specific daily cpm, VM counts/day, and steps/day were all higher for wrist-worn accelerometers. Wrist VM counts/day were approximately 5 times greater and step counts double that of the hip worn accelerometers for the same measure at each time point. The correlation between each of the five measures for hip- and wrist-worn accelerometers displayed moderate to excellent agreement (Pearson's *r* = 0.59 to 0.84). Overall, the correlations between hip and wrist accelerometers were lower during T2, higher at T3, and were highest at postpartum.

**Table 2:** Daily mean values and Pearson's correlations between hip and wrist accelerometer measures, during pregnancy and postpartum.

			Wrist				
	T2 (*n* = 31)		Axis 1 cpm	Axis 2 cpm	Axis 3 cpm	Vector magnitude counts/day	Steps/day
		Daily mean total (sd)	16,855.44 (6695.2)	17,309.8 (6273.5)	20,608.18 (7726.7)	1,595,614.0 (467,866.4)	7085.74 (1979.3)
Hip	Axis 1 cpm	2927.4 (1312.7)	0.59	0.57	0.56	0.58	0.71
	Axis 2 cpm	3247.1 (1544.9)	0.70	0.68	0.66	0.68	0.76
	Axis 3 cpm	4020.5 (1907.9)	0.67	0.64	0.62	0.65	0.71
	Vector magnitude counts/day	290,195.0 (102,539.8)	0.70	0.68	0.66	0.68	0.78
	Steps/day	3654.0 (1598.9)	0.96	0.94	0.78	0.78	0.77


			Wrist				
	T3 (*n* = 62)		Axis 1 cpm	Axis 2 cpm	Axis 3 cpm	Vector magnitude counts/day	Steps/day
		Daily mean value (sd)	15,339.0 (5544.4)	15,870.8 (5148.7)	18,822.1 (6897.9)	1,751,923.0 (608,144.9)	7554.2 (2424.7)
Hip	Axis 1 cpm	2572.52 (1089.3)	0.64	0.59	0.56	0.60	0.62
	Axis 2 cpm	2538.979 (950.5)	0.73	0.68	0.72	0.72	0.76
	Axis 3 cpm	3256.887 (1373.2)	0.79	0.73	0.75	0.77	0.77
	Vector magnitude counts/day	301,063.4 (112,410.1)	0.82	0.76	0.77	0.79	0.80
	Steps/day	3522.94 (1286.3)	0.67	0.59	0.59	0.62	0.74


			Wrist				
	Postpartum^a^ (*n* = 55)		Axis 1 cpm	Axis 2 cpm	Axis 3 cpm	Vector magnitude counts/day	Steps/day
		Daily mean value (sd)	18,457.6 (7361.5)	18,778.0 (6923.7)	22,392.7 (8249.3)	2,083,842.8 (776,000.3)	9208.7 (3254.3)
Hip	Axis 1 cpm	3289.7 (1427.8)	0.72	0.73	0.71	0.73	0.75
	Axis 2 cpm	3784.3 (1690.1)	0.80	0.80	0.77	0.80	0.80
	Axis 3 cpm	4680.6 (2032.2)	0.84	0.84	0.81	0.84	0.80
	Vector magnitude counts/day	421,463.6 (174,410.1)	0.83	0.83	0.80	0.83	0.84
	Steps/day	4642.2 (2056.2)	0.80	0.78	0.77	0.79	0.84

T2: trimester 2; T3: trimester 3; PP: postpartum; cpm: counts per minute; sd: standard deviation; a: aggregated for women providing at least one time point during postpartum period.

When VM counts/day were categorized into quartiles, hip- and wrist-worn accelerometers classified participants into the same quartile 86.1% of the time (weighted Kappa of 0.67 (95% C·I 0.62–0.72)) during pregnancy and 87.1% of the time (weighted Kappa of 0.68 (95% C·I 0.62–0.71)) postpartum. Percentage agreement and weighted kappa statistics were similar for steps/day: during pregnancy, hip- and wrist-worn accelerometers classified participants into the same quartile 85.4% of the time (weighted Kappa of 0.65 (95% C·I 0.56–0.66)) and postpartum 89.5% of the time (weighted Kappa of 0.75 (95% C·I 0.71–0.77)). Participants classified in the highest quartile for VM counts/day and steps/day by hip were never classified into the lowest quartiles for wrist and vice versa. Bland-Altman plots indicated the proportional bias comparing hip- to wrist-worn accelerometry. The mean difference between hip and wrist-worn accelerometers was −3908.8 (Limits of Agreement (LOA): −7231.8, −585.8) for VM counts during pregnancy and −4327.3 (−7821.7, −832.8) postpartum, and −1,427,619 for steps/day (−2.436,079.8, −419,158.2) during pregnancy and −1,604,897.6 (−2.726,118.4, −483,676.7) postpartum (Appendix 1).

### 3.3 Change in physical activity levels

**Fig. 3:**
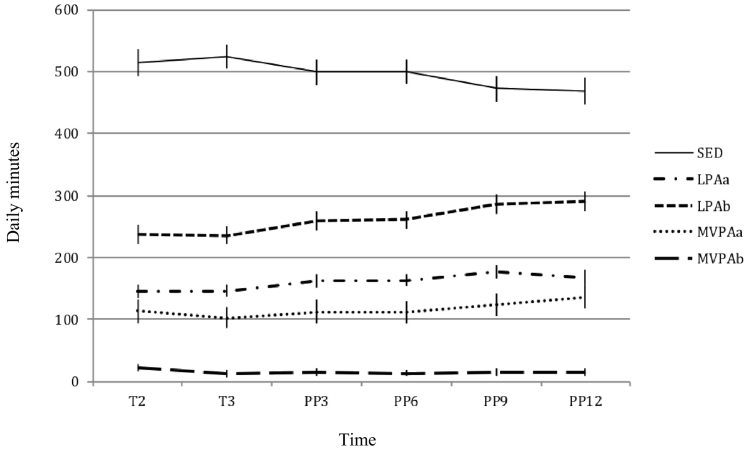
Change in sedentary behavior and physical activity during pregnancy and postpartum in the Nurture study (North Carolina, 2014–2015, *n* = 43). SED: sedentary; LPA: light physical activity; MVPA: moderate-vigorous physical activity; a: mean daily activity derived using the Swartz cut-points; b: mean daily activity derived using the Troiano cut-points.

A total of 43 women provided valid physical activity data for at least one time point during pregnancy and one time point postpartum. Based on hip-derived cpm, women engaged in approximately 500 min of sedentary time each day during T2, T3, and early postpartum (Fig. 3; Appendix 2). This decreased somewhat at 9- and 12-months postpartum. The total daily minutes women spent in physical activity was consistent regardless of cut point used, but the classification of LPA and MVPA differed as expected. For example, according to Troiano cutpoints, women predominantly engaged in LPA and achieved relatively little MVPA [e.g., T2: LPA: 235.9 (SD: 75.3) minutes, MVPA: 24.2(48.1); PP3: LPA: 259.6(112.9), MVPA:10.0(8.2); PP9: LPA: 285.5(111.9), MVPA: 10.8 (8.7)]. In contrast, the ratio of LPA to MVPA was significantly lower using the Swartz cut points [e.g., T2: LPA: 148.6(40.8), MVPA: 111.5(97.9); PP3: LPA: 164.5(67.3), MVPA: 105.1(62.0); PP9: LPA: 176.5(64.5), MVPA: 119.7(73.8)].

**Fig. 4:**
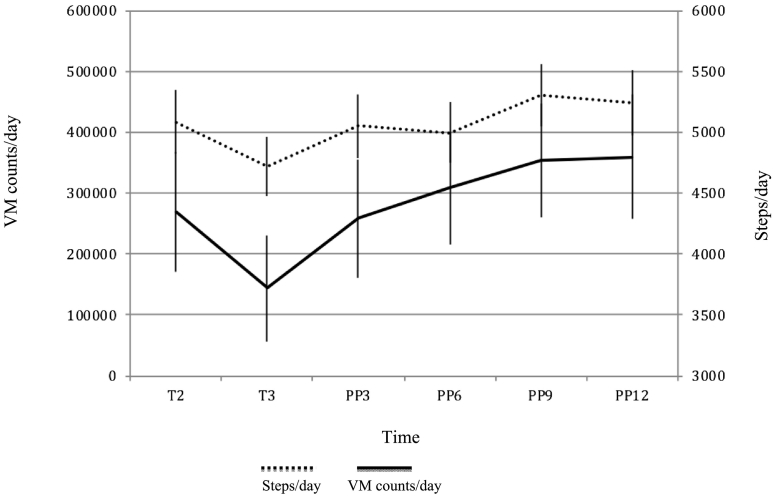
Change in vector magnitude (VM) counts/day and steps/day during pregnancy and postpartum in the Nurture study (North Carolina, 2014–2015, *n* = 43).

Overall, women's sedentary time did not differ between T2 and T3 (Table 3; Fig. 3). Women were however significantly less sedentary in the postpartum period compared to T3 [PP3: −23.8 (−42.2, −5.3) minutes; PP6: −22.8 (−40.5, −5.2); PP9: −48.8 (−66.5, −31.0); PP12: −54.0 (−73.5, −34.6)] (and T2, Appendix 3). They engaged in significantly more VM counts/day and steps/day during T2 and postpartum compared to T3 (Fig. 4). Again, change in LPA and MVPA during pregnancy and postpartum differed depending on the cut points used. In general, women engaged in lower levels of MVPA and higher levels of LPA postpartum. Compared to T3, this increased significantly at T9 and T12 for the former, and throughout the postpartum period for the latter (Table 3). Overall, women were significantly more active and less sedentary in PP9 and PP12, compared to pregnancy and early postpartum [e.g. PP3 as baseline: PP9: sedentary: −25.0 (−44.7, −5.2), LPA_(Swartz)_: 12.6 (0.74,24.4), LPA_(Troiano)_: 24.1 (5.5,42.7); PP12: sedentary: −30.3(−51.6, −9.0), LPA_(Swartz):_ 24.1 (5.5,42.7), LPA_(Troiano)_: 29.7 (9.6,49.8) (Appendix 4)] but there were no significant differences in VM and step counts compared to T2.

**Table 3:** Longitudinal changes in daily physical activity during pregnancy and postpartum (*n* = 43).

	Daily physical activity ß [95% confidence intervals]					
	T2	T3	PP3	PP6	PP9	PP12
Physical activity (minutes)						
SED	−12.6	B A S E L I N E	−23.8^⁎^	−22.8^⁎^	−48.8^⁎⁎⁎^	−54.0^⁎⁎⁎^
	[−31.6,6.5]		[−42.2,−5.3]	[−40.5,−5.2]	[−66.5,−31.0]	[−73.5,−34.6]
LPA^a^	1.9		15.4^⁎⁎^	17.0^⁎⁎^	28.0^⁎⁎⁎^	22.4^⁎⁎⁎^
	[−9.4,13.3]		[4.4,26.4]	[6.5,27.5]	[17.3,38.6]	[10.8,34.1]
MVPA^a^	10.8		8.1	6.5	20.7^⁎⁎⁎^	31.5^⁎⁎⁎^
	[−1.7,23.3]		[−3.9,20.2]	[−5.1,18.0]	[9.1,32.3]	[18.8,44.3]
LPA^b^	4.3		22.0^⁎^	22.8^⁎⁎^	46.1^⁎⁎⁎^	51.7^⁎⁎⁎^
	[−13.6,22.2]		[4.6,39.4]	[6.2,39.4]	[29.4,62.8]	[33.3,70.0]
MVPA^b^	8.0^⁎⁎⁎^		1.5	0.40	2.4	2.2
	[3.7,12.3]		[−3.1,6.1]	[−3.6,4.4]	[−1.6, 6.4]	[−2.2,6.6]
Vector magnitude counts	72,150.2^⁎⁎⁎^		65,334.6^⁎⁎⁎^	59,575.2^⁎⁎^	1.1e + 05^⁎⁎⁎^	1.1e + 05^⁎⁎⁎^
	[32,626.9,1.1e + 05]		[27,153.5,1.0e + 05]	[23,043.3,96,107.2]	[77,858.0,1.5e + 05]	[64,721.3,1.5e + 05]
Step counts	550.0^⁎^		507.4^⁎^	749.7^⁎⁎⁎^	973.8^⁎⁎⁎^	997.1^⁎⁎⁎^
	[117.7982.3]		[88.8926.0]	[349.4,1150.0]	[571.0,1376.6]	[555.1,1439.1]

SED: sedentary; LPA: light physical activity; MVPA: moderate-vigorous physical activity; a: mean daily activity derived using the Swartz cut-points; b: mean daily activity derived using the Troiano cut-points; T2: Trimester 2; T3: Trimester 3; PP3: 3 months postpartum; PP6: 6 months postpartum; PP9: 9 months postpartum; PP12: 12 months postpartum; models include women with at least 3 valid days of physical activity data during both pregnancy and postpartum; adjusted for daily wear time; ^⁎^*p* < 0.05, ^⁎⁎^*p* < 0.01, ^⁎⁎⁎^*p* < 0.001.

## 4 Discussion

To our knowledge, this is one of the first studies to assess objectively measured physical activity and sedentary behavior during pregnancy and postpartum using hip- and wrist-worn accelerometers concurrently, focusing on a sample of low-income, predominantly Black or African-American US women. Compliance was higher for wrist-worn compared to hip-worn accelerometers over the course of the measurement period. Wrist-worn accelerometers provided a greater number of hours per day and days per measurement period of valid physical activity data, but also a greater number of counts for each outcome compared to hip-worn accelerometers. Overall, the convergent validity between hip and wrist accelerometers was higher during late pregnancy and during the postpartum period. We observed moderate to substantial correlations between hip- and wrist-worn axis-specific cpm, steps/day, and VM counts/day; weighted kappa statistics showed moderate to excellent agreement between measures. Using hip-derived physical activity levels, sedentary and active time differed little between T2 and T3, but light physical activity levels increased and sedentary time decreased postpartum.

To date, hip-worn accelerometers have tended to be used to obtain objectively measured physical activity and sedentary behavior, for which a range of valid algorithms and cut points exist to derive physical activity intensity and physical activity energy expenditure (Troiano et al., 2014). However more recently, there has been a move toward use of wrist-worn accelerometers, in part as they appear to ensure better compliance with wear protocols in both adults (Troiano et al., 2014) and children (Fairclough et al., 2015). As hypothesized, the compliance was better for wrist-worn accelerometers, providing greater volumes of valid accelerometry data during both a measurement day and week. Hip-worn accelerometers may become more problematic in the latter stages of pregnancy when wearing an accelerometer could become uncomfortable. Indeed, a previous study found that compliance with hip-worn accelerometers declined from 90% during early to 47% during late pregnancy (Rousham et al., 2006).

Given that pregnancy-specific physical activity cut points do not exist (Evenson et al., 2012b), and placement of hip-worn accelerometers may be increasingly altered during pregnancy, it is possible that derived physical activity levels obtained later in pregnancy may be subject to greater error. Wrist-worn accelerometers, which should remain in the same place regardless of changes during pregnancy, may be more likely to provide consistency across measurement time points. This said, wrist-worn accelerometers do capture movement of the whole body, including movement of the trunk in the absence of the whole body, as suggested by consistently higher recorded counts for wrist-derived outcomes here. Nevertheless, subjective or self-reported measures tend to result in over-estimation of physical activity in adults (Adamo et al., 2009), and agreement between questionnaire derived physical activity levels and objective measures (i.e. pedometers and accelerometers) in pregnant women is only slight to fair (Evenson et al., 2012a). In order to ensure accurate assessment of physical activity during pregnancy and postpartum, greater use of objective methods, and of accelerometers in particular, is therefore warranted (Guérin et al., 2018). This work suggests that wrist-worn accelerometers are both feasible for measurement of physical activity during pregnancy and postpartum (van Hees et al., 2011), and may be preferable for pregnant women in terms of comfort and compliance.

Currently, there are fewer developed processing protocols for wrist-worn accelerometers (Mannini et al., 2013). As described previously (Hildebrand et al., 2014; Kamada et al., 2016; Tudor-Locke et al., 2015), wrist-worn accelerometers result in greater axis-specific counts/min, total number of steps/day, and VM counts/day in comparison to hip-worn accelerometers. It has been suggested that these differences may in part be due to differing biomechanics at wrist and hip sites (Kamada et al., 2016), which change as individuals move from childhood (Tudor-Locke et al., 2015) into adulthood (Hildebrand et al., 2014; Kamada et al., 2016). Although biomechanical differences are also likely to occur over the course of pregnancy, compared to early pregnancy, higher correlations between hip- and wrist-worn accelerometers in both late pregnancy and postpartum were observed here. The overall correlation between wrist- and hip-worn accelerometers was moderate to substantial, as seen previously in samples of older US women (Kamada et al., 2016) and Norwegian adults (Hildebrand et al., 2014). Kappa statistics also indicated moderate to excellent agreement, suggesting that these measures were able to appropriately classify individual women's physical activity for both VM counts/day and steps/day.

It should be noted that several studies have reported better classification accuracy between hip- and wrist-worn accelerometers at higher and lower intensities of physical activity (Hildebrand et al., 2014; Kamada et al., 2016). Treadmill studies suggest there may be a plateauing of counts at higher intensities to account for this (John et al., 2012), but this does not appear to be the case in free-living or non-laboratory-based studies (Barnett et al., 2015; Vanhelst et al., 2009); at very low activity intensities, the Choi algorithm was used here to distinguishing non-wear time from sedentary time (Choi et al., 2011). As reported previously (Hildebrand et al., 2014), wrist and hip accelerometers therefore appear to provide comparable estimates of physical activity and sedentary behavior and have been validated widely for this use (Hildebrand et al., 2014). Further work is now required to determine how physiological differences that occur during pregnancy influence activity processing and detection in women during the transition to motherhood. However, ensuring that this information is gained from high-quality research studies using objective measures will advance the research field whilst ensuring women can be confident in recommendations made for physical activity during pregnancy and postpartum.

As no agreed processing criteria exist to derive activity intensity for wrist-worn accelerometers (Kamada et al., 2016), a measure of women's physical activity levels during pregnancy and postpartum using only hip-worn accelerometers and existing validated cut points was derived (Swartz et al., 2000; Troiano et al., 2008), and used to assess longitudinal change over time. As expected based on previous work in US women (Evenson and Wen, 2011; Hesketh and Evenson, 2016), participants were sedentary for a large proportion of the measurement day (500 min or ~8.3 h), regardless of time point (i.e., 2 points during pregnancy or 4 postpartum). Overall, women's physical activity during pregnancy in this study was slightly lower than that observed in a small sample of Australian women (*n* = 30) during T3 (Harrison et al., 2011), and slightly higher than that of another UK-based sample of women (*n* = 97) (Currie et al., 2015). Both studies used Freedson cutpoints (Freedson et al., 1998) to assess physical activity, finding Australian women engaged in 353 min of LPA and 50 min of MPA on average per day in T3, whereas UK women accrued 115 and 30 min of LPA and MVPA respectively during pregnancy (Evenson et al., 2012a). In the latter, as here, MVPA decreased during pregnancy (Currie et al., 2015).

There is a general trend for MVPA to decrease during pregnancy (Currie et al., 2015) and be replaced by LPA postpartum, which may in part reflect the women's changes in activity type during the transition to motherhood (Borodulin et al., 2008). Yet despite this, levels of MVPA in this group of low-income women appear to be approximately half those of a similar sample of women from North Carolina postpartum (Evenson et al., 2012b). Although increases in physical activity did occur toward the end of the first year post-birth here, most health benefits garnered from physical activity appear to be associated with MVPA rather than with lighter intensity activity (Department of Health, 2011). It may therefore be that women, and especially those from lower-income settings, require additional support to maintain, begin to engage or re-engage in, moderate levels of physical activity as they enter the postpartum period.

### 4.1 Strengths and limitations

This is one of the first studies to compare objectively measured physical activity and sedentary behavior using hip- and wrist-worn accelerometers during pregnancy and the postpartum period using women sampled from a larger cohort study. We used a previously established protocol (Kamada et al., 2016), assessing physical activity outputs common to both accelerometer types. This negated the need for wrist-worn intensity cut points, for which no consensus currently exists. The accelerometer wear-protocol women were instructed to follow may have resulted in differences in compliance; we matched hip- and wrist-worn data hour for hour ensuring a like-for-like comparison, and minimized potential bias by excluding hours between 12 pm–6 am for both types of accelerometer. This also allowed us to account for time when women were likely to be sleeping in wrist-worn accelerometers but had removed their hip-worn accelerometers.

To examine changes in physical activity during pregnancy and postpartum, we used two common cut points (Swartz et al., 2000; Troiano et al., 2008) (during pregnancy) to derive hip-worn daily physical activity levels, which also allowed for comparison of how accelerometer cut points influenced our findings. Aggregating women's minutes spent sedentary, in LPA and MVPA at the daily level, hierarchical regression analyses were conducted to determine how activity at each time point (level 1) clustered within women (level 2) changed over time. This increased our power to detect an association. Although our sample size was relatively small, it is comparable to other samples assessing objectively measured physical activity during pregnancy and postpartum (Currie et al., 2015; Evenson et al., 2012b; Harrison et al., 2011). The women recruited in the sub-study were largely representative of the low-resource predominantly Black and African American US women participating in the larger Nurture study; those providing data during pregnancy and postpartum were also similar to those originally recruited into the sub-study. Though the absolute amount of physical activity and sedentary behavior measured here may not be generalizable to other populations, the general findings relating to device agreement should not differ considerably.

## 5 Conclusions

The correlation between hip- and wrist-worn accelerometers was moderate to substantial throughout pregnancy and postpartum, suggesting that either form of measurement is suitable for activity assessment during this period. However, compliance with wrist-worn (compared to hip-worn) accelerometers appears to be higher, and wrist-worn accelerometers may be preferable to women due to physical changes they undergo during pregnancy and postpartum. As physical activity levels, and MVPA in particular, appear to decline during later pregnancy and do not appear to rebound quickly after birth, women may benefit from additional support to safely reintroduce moderate levels of physical activity earlier in the postpartum period.

## Conflict of interest

The authors declare no conflicts of interest.

## Appendix A Supplementary data

Supplementary material Image 1
