# Supplementary material for: Physical activity and sedentary behavior during pregnancy and postpartum, measured using hip and wrist-worn accelerometers
Source: Prev Med Rep. 2018 Apr 19;10:337–45. doi: 10.1016/j.pmedr.2018.04.012 (PMC5984239; doi:10.1016/j.pmedr.2018.04.012)
Supplement: Supplementary file 1 — Supplementary material [file mmc1.docx]

**Appendix 1a: Bland Altman plot showing the (limits of) agreement between hip- and wrist-derived VM counts/day during pregnancy (North Carolina, 2014-2015; n=87)**

Difference between hip and wrist VM counts/day

Average of hip and wrist VM counts/day

**Appendix 1b: Bland Altman plot showing the (limits of) agreement between hip- and wrist-derived VM counts/day accelerometers during postpartum (North Carolina, 2014-2015; n=55)**

Difference between hip and wrist VM counts/day

Average of hip and wrist VM counts/day

**Appendix 1c: Bland Altman plot showing the (limits of) agreement between hip- and wrist-derived steps/day during pregnancy (North Carolina, 2014-2015; n=87)**

Average of hip and wrist step counts

Difference between hip and wrist steps/day

Average of hip and wrist steps/day

**Appendix 1d: Bland Altman plot showing the (limits of) agreement between hip- and wrist-derived steps/day during postpartum (North Carolina, 2014-2015; n=55)**

Average of hip and wrist steps/day

Difference between hip and wrist steps/day

| **Appendix 2: Daily mean physical activity levels measured using hip-worn monitors, by time of measurement** | | | | | | | | | | | | |
| --- | --- | --- | --- | --- | --- | --- | --- | --- | --- | --- | --- | --- |
|  | T2  (n=34) | | T3  (n=64) | | PP3  (n=30) | | PP6  (n=37) | | PP9  (n=33) | | PP12  (n=35) | |
|  | Mean | sd | Mean | sd | Mean | sd | Mean | sd | Mean | sd | Mean | sd |
| **Wear time** | | | | | | | | | | | | |
| Valid days | 6.5 | 1.7 | 6.2 | 1.8 | 5.7 | 1.7 | 5.3 | 1.6 | 6.0 | 2.1 | 5.6 | 1.6 |
| Hours/day | 12.6 | 2.2 | 12.3 | 2.1 | 13.2 | 2.2 | 13.1 | 1.6 | 12.8 | 1.9 | 13.3 | 1.6 |
|  |  |  |  |  |  |  |  |  |  |  |  |  |
| **Daily physical activity (minutes/day)** | | | | | | | | | | | | |
| Sedenatry | 493.8 | 107.8 | 503.6 | 103.7 | 517.0 | 119.0 | 502.1 | 98.8 | 465.4 | 114.0 | 485.7 | 93.0 |
| Trioano^a^ |  |  |  |  |  |  |  |  |  |  |  |  |
| Light | 235.9 | 75.3 | 223.6 | 71.0 | 259.6 | 112.9 | 267.1 | 61.8 | 285.5 | 111.9 | 296.9 | 90.5 |
| Moderate | 23.9 | 47.1 | 8.0 | 9.0 | 10.0 | 8.0 | 12.9 | 11.2 | 10.6 | 8.7 | 12.7 | 14.0 |
| Vigorous | 0.36 | 1.1 | 0.03 | 0.11 | 0.04 | 0.2 | 0.11 | 0.27 | 0.17 | 0.75 | 0.07 | 0.17 |
| MVPA | 24.2 | 48.1 | 8.1 | 9.0 | 10.0 | 8.2 | 13.0 | 11.3 | 10.8 | 8.7 | 12.8 | 14.0 |
| Swartz^b^ |  |  |  |  |  |  |  |  |  |  |  |  |
| Light | 148.6 | 40.8 | 142.4 | 43.5 | 164.5 | 67.3 | 172.2 | 40.1 | 176.5 | 64.5 | 176.2 | 42.6 |
| Moderate | 110.2 | 94.7 | 89.2 | 41.1 | 105.0 | 62.0 | 107.6 | 38.6 | 119.4 | 73.9 | 133.4 | 69.2 |
| Vigorous | 1.3 | 3.4 | 0.07 | 0.17 | 0.12 | 0.32 | 0.35 | 0.71 | 0.34 | 1.20 | 0.14 | 0.25 |
| MVPA | 111.5 | 97.9 | 89.3 | 41.1 | 105.1 | 62.0 | 108.0 | 38.5 | 119.7 | 73.8 | 133.5 | 69.2 |
|  |  |  |  |  |  |  |  |  |  |  |  |  |
| **Average daily physical activity** | | | | | | | | | | | | |
| VM Counts/day | 433698.8 | 401078.1 | 294405.4 | 113029.4 | 382779.7 | \| 185624.9 \| \| --- \| | 404877.7 | \| 112232.5 \| \| --- \| | 434314.4 | 216553.3 | 448255.5 | \| 190085.4 \| \| --- \| |
| Step Counts/day | 3984.0 | 1816.8 | 3455.5 | 1293.8 | 4111.5 | 2009.9 | 4522.1 | 1738.3 | 4867.0 | 2368.6 | 4859.9 | 2048.7 |

T2: Trimester 2; T3: Trimester 3; PP3: 3 months postpartum; PP6: 6 months postpartum; PP9: 9 months postpartum; PP12: 12 months postpartum; a: mean daily activity derived using the Swartz cut-points; b: mean daily activity derived using the Troiano cut-points.

|  | | T2 | PP3 | PP6 | PP9 | PP12 |  |
| --- | --- | --- | --- | --- | --- | --- | --- |
|  | | Daily physical activity ß[95% confidence intervals] | | | | | |
| **Physical activity (minutes/day)** | |  |  |  |  |  |  |
| SED |  | B a s e l i n e | -11.2 | -10.2 | -36.2^***^ | -41.4^***^ |  |
|  |  |  | [-32.1,9.7] | [-30.0,9.6] | [-56.6,-15.7] | [-63.5,-19.4] |  |
| LPA^a^ |  |  | 13.5^*^ | 15.1^*^ | 26.0^***^ | 20.5^**^ |  |
|  |  |  | [1.0,25.9] | [3.2,26.9] | [13.8,38.2] | [7.3,33.6] |  |
| MVPA^a^ |  |  | -2.6 | -4.3 | 9.9 | 20.8^**^ |  |
|  |  |  | [-16.3,11.1] | [-17.3,8.7] | [-3.5,23.4] | [6.3,35.2] |  |
| LPA^b^ |  |  | 17.7 | 18.5 | 41.8^***^ | 47.4^***^ |  |
|  |  |  | [-1.9,37.4] | [-0.1,37.2] | [22.5,61.0] | [26.6,68.1] |  |
| MVPA^b^ |  |  | -6.4^**^ | -7.6^***^ | -5.6^*^ | -5.7^*^ |  |
|  |  |  | [-11.1,-1.7] | [-12.0,-3.1] | [-10.2,-1.0] | [-10.7,-0.8] |  |
|  |  |  |  |  |  |  |  |
| **Vector magnitude counts/day** | |  | -6815.6 | -1.3e+04 | 42463.3^*^ | 32889.8 |  |
|  |  |  | [-5.0e+04,36449.2] | [-5.4e+04,28446.28] | [41.1,84885.5] | [-1.3e+04,78611.0] |  |
| **Step counts/day** |  |  | -42.5 | 199.7 | 423.9 | 447.1 |  |
|  |  |  | [-516.3,431.2] | [-249.9,649.3] | [-40.4,888.1] | [-53.5,947.7] |  |
|  |  |  |  |  |  |  |  |

**Appendix 3: Longitudinal changes in daily physical activity postpartum in the PAiP sample (North Carolina, 2014-2015; n=43; T2 as baseline)**

SED: Sedentary; LPA: Light physical actvitiy; MVPA: Moderate-vigorous physical activity; a: mean daily activity derived using the Swartz cut-points; b: mean daily activity derived using the Troiano cut-points; T2: Trimester 2; T3: Trimester 3; PP3: 3 months postpartum; PP6: 6 months postpartum; PP9: 9 months postpartum; PP12: 12 months postpartum; odels include women with at least 3 valid days of physical activity data during both pregnancy and postpartum; adjusted for daily wear time; ^*^ *p* < 0.05, ^**^ *p* < 0.01, ^***^ *p* < 0.001

**Appendix 4: Longitudinal changes in daily physical activity postpartum in the PAiP sample (North Carolina, 2014-2015; n=43; PP3 as baseline**

|  | | PP3 | PP6 | PP9 | PP12 |  |
| --- | --- | --- | --- | --- | --- | --- |
|  | | Daily physical activity ß[95% confidence intervals] | | | | |
| **Physical activity (minutes/day)** | |  |  |  |  |  |
| SED |  | B a s e l i n e | 0.94 | -25.0^*^ | -30.3^**^ |  |
|  |  |  | [-18.6,20.5] | [-44.7,-5.2] | [-51.6,-9.0] |  |
| LPA^a^ |  |  | 1.6 | 12.6^*^ | 7.0 |  |
|  |  |  | [-10.1,13.3] | [0.74,24.4] | [-5.7,19.8] |  |
| MVPA^a^ |  |  | -1.7 | 12.6 | 23.4^**^ |  |
|  |  |  | [-14.5,11.1] | [-0.36,25.5] | [9.4,37.4] |  |
| LPA^b^ |  |  | 0.82 | 24.1^*^ | 29.7^**^ |  |
|  |  |  | [-17.6,19.2] | [5.5,42.7] | [9.6,49.8] |  |
| MVPA^b^ |  |  | -1.1 | 0.83 | 0.70 |  |
|  |  |  | [-5.5,3.3] | [-3.6,5.7] | [-4.1,5.5] |  |
|  |  |  |  |  |  |  |
| **Vector magnitude counts/day** | |  | -5759.4 | 49278.8^*^ | 39705.4 |  |
|  |  |  | [8371.8,90185.9] | [-4438.4,83849.1] | [-8711.1,85782.4] |  |
| **Step counts/day** |  |  | 242.3 | 466.4^*^ | 489.7^*^ |  |
|  |  |  | [-201.3,685.8] | [17.9,914.8] | [5.7,973.6] |  |
|  |  |  |  |  |  |  |

SED: Sedentary; LPA: Light physical actvitiy; MVPA: Moderate-vigorous physical activity; a: mean daily activity derived using the Swartz cut-points; b: mean daily activity derived using the Troiano cut-points; T2: Trimester 2; T3: Trimester 3; PP3: 3 months postpartum; PP6: 6 months postpartum; PP9: 9 months postpartum; PP12: 12 months postpartum; odels include women with at least 3 valid days of physical activity data during both pregnancy and postpartum; adjusted for daily wear time; ^*^ *p* < 0.05, ^**^ *p* < 0.01, ^***^ *p* < 0.0001
